# In Too Deep: A Point-of-Care Ultrasound (POCUS) Escape Room

**DOI:** 10.21980/J8.52100

**Published:** 2025-10-31

**Authors:** Brandon M Wubben

**Affiliations:** * University of Iowa, Department of Emergency Medicine, Iowa City, IA

## Abstract

**Audience:**

Emergency medicine residents and emergency ultrasound fellows.

**Introduction:**

Point-of-care ultrasound (POCUS) is an essential emergency medicine skill that requires hands-on practice and an understanding of anatomy in three-dimensional space.^1^ Experientially, some common POCUS challenges are identifying foreign bodies in soft tissue, recognizing nerves, and identifying lower extremity veins in relation to other anatomic landmarks. But finding novel ways to challenge and engage advanced learners who have mastered basic POCUS content can be difficult, and this was the impetus of the current gamified educational activity.

**Educational Objectives:**

By the end of this session, the participant will be able to:

1. Evaluate and identify the nature of metallic foreign bodies using POCUS.
2. Identify common emergency department fractures on X-Ray and identify relevant sonoanatomy for ultrasound-guided regional anesthesia applications relevant to those fractures.
3. Identify normal lower extremity venous POCUS sonoanatomy and demonstrate understanding of proximal versus distal anatomical location within the lower extremity venous system.

**Educational Methods:**

A hands-on, gamified approach was used. This approach builds on previously published escape room models to focus on the application of the three POCUS indications described above.^2^,^3^

**Research Methods:**

After the activity, participants filled out a standardized teaching evaluation including questions about the quality of the material presented, the degree to which they felt actively involved as learners, and free-text qualitative feedback.

**Results:**

Our test group including emergency ultrasound fellows and senior emergency medicine residents successfully completed the escape room. Three of the four participants (75%) completed the evaluation; 3/3 (100%) rated the escape room as exceeded expectations for actively involving learners and qualitatively reported that the activity was “fun,” “interactive,” “engaging,” and “innovative.”

**Discussion:**

We found that using an escape room format for POCUS education was effective and engaging. However, it is important to note that small details in the escape room design may have large impacts on the ability of the learners to complete the activity and meet the educational goals. Overall, we found that this activity was effective and fun for both learners and educators.

**Topics:**

Point-of-care ultrasound (POCUS), team building, foreign body identification, ultrasound image review, lower extremity venous, ultrasound-guided regional anesthesia.

## USER GUIDE

**Table:** 

List of Resources:	
Abstract	50
User Guide	52
Small Groups Learning Materials	54
Appendix A: Small Group Application Exercise	54
Appendix B: Handout	65

**Learner Audience:**

Senior Residents, Emergency Ultrasound Fellows

**Time Required for Implementation:**

The session leader will need to order or gather supplies in advance (approximately one to two weeks). The setup can be completed almost entirely prior to the session, with about 20 minutes needed on the day of the session to place items in the room. The session is designed to take about 50 minutes, plus 10 minutes for debriefing, for a total of one hour. If multiple groups are rotating through the room, about 15 minutes is needed to reset the game before the next group starts.

**Recommended Number of Learners Per Instructor:**

The escape room could reasonably be executed with three to six learners depending on the size of the room used and was initially tested with four. If there are more than six learners, the author recommends combining the session with other rotating small group activities rather than trying to run multiple rooms at once due to resource intensity and material costs.

**Topics:**

Point-of-care ultrasound (POCUS), team building, foreign body identification, ultrasound image review, lower extremity venous anatomy, ultrasound-guided regional anesthesia.

**Objectives:**

By the end of this escape room learners will be able to:

1. Evaluate for and identify the nature of metallic foreign bodies using POCUS.
2. Identify common emergency department fractures on X-Ray and identify relevant sonoanatomy for ultrasound-guided regional anesthesia applications relevant to those fractures.
3. Identify normal lower extremity venous POCUS sonoanatomy and demonstrate understanding of proximal versus distal anatomical location within the lower extremity venous system.

### Linked objectives and methods

In our experience, and in the prior literature, hands-on learning using gamification is an effective and meaningful experience for the learner.^4^ We wanted to take topics that are often presented in a lecture format and make them interactive and hands-on, prompting learners to engage with the material and interact with each other. To accomplish this, the escape room was designed with three distinct phases or clues, each targeted at a different objective.

Phase one presents learners with a foreign body embedded in gelatin, requiring them to identify both the presence and nature of the foreign body (objective 1). Learners should recognize that the composition of the foreign body affects its ultrasonographic appearance, and this can be used to determine the identity of the foreign body. Metallic bodies such as a coin cast a dense posterior shadow, and their length can be measured. Phase two is a compound task that presents learners with common emergency department fractures requiring identification based on X-Rays and secondarily requires identification of regional anesthesia techniques that would provide analgesia for the relevant injury. Learners must identify sonographic anatomy to determine what regional anesthesia target is being presented (objective 2). Finally, clue three asks learners to identify the normal sonographic appearance of the confluences of the lower extremity venous system and place them in order of their anatomical proximal versus distal location (objective 3).

### Recommended pre-reading for facilitator

- Costantino TG, Goett HJ. Deep vein thrombosis. In: *Ma and Mateer’s Emergency Ultrasound*, 4^th^ ed. McGraw-Hill Education; 2021. Accessed February 13, 2024.
- Byars DV, Martel ML, Noble M. Ultrasound-guided regional anesthesia. In: *Ma and Mateer’s Emergency Ultrasound*, 4^th^ ed. McGraw-Hill Education; 2021. Accessed February 13, 2024.

### Small group application exercise (sGAE)

See the following attached materials for this small group exercise

- Appendix A: sGAE
- Appendix B: Handout
- 2CLUE2.pptx
- CLUE3a.pptx
- CLUE3b.PUZZLED8.docx

### Results

Our test group comprised two emergency ultrasound fellows and two senior residents who were able to successfully complete the escape room with several hints. Participants completed an institutionally-standardized teaching evaluation asking about items including the quality of the material presented, the degree to which they felt actively involved as learners, and free-text qualitative feedback. Standardized questions utilized a Likert scale with three options: “Below expectations,” “Met expectations,” and “Exceeded expectations.” Of the three participants who completed the evaluation (75%), 3/3 (100%) rated the escape room as ‘exceeded expectations’ for actively involving learners and qualitatively reported that the activity was “fun,” “interactive,” “engaging,” and “innovative.”

### Tips for Successful Implementation

1. **Important:** This escape room as designed and tested is meant for learners familiar with escape room mechanics. The author strongly recommends starting the activity with the facilitator opening Clue 2 (skipping Clue 1 entirely) if the learners or facilitator are not experienced because of the added time it would take for novice learners to learn the escape room mechanics before making progress on the clues. The preparation required is also much shorter if omitting Clue 1.
2. Don’t let learners get stuck without a hint for too long. Asking “do you want a hint?” if they seem stuck or you know they are far off track will prompt them to make a decision about what they want to do next, even if they end up saying no to a hint.
3. If you as the facilitator made any mistake in preparing the escape room, just give the learners the correct prompt or clue to let them move on once you get there. The structure of the clues is meant to encourage learning engagement, not cause distraction.

## Supplementary Information









## Figures and Tables

**Figure 1A, 1B, 1C:**
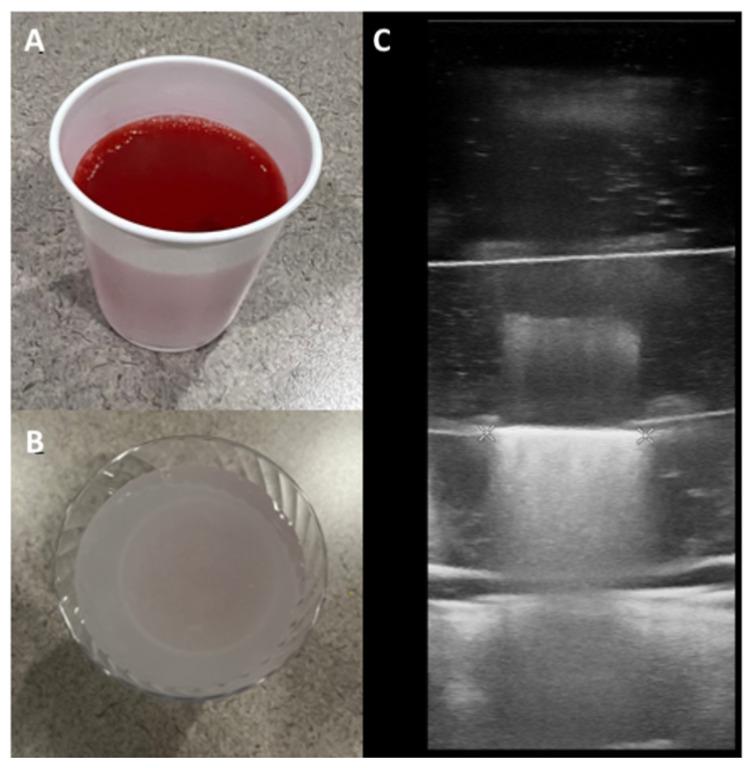
Example container (1A) filled with opaque gelatin and containing a coin. Note the gelatin does not extend all the way to the top of the cup. As shown in 1B, the cup can be placed in a larger cup of cold water to create a water bath (cold water should be provided instead of warm water to avoid melting the gelatin). Using a linear probe in the water bath, the coin is visible within the gel and the size can be measured (1C). Author’s own photograph and image.

**Figure:**
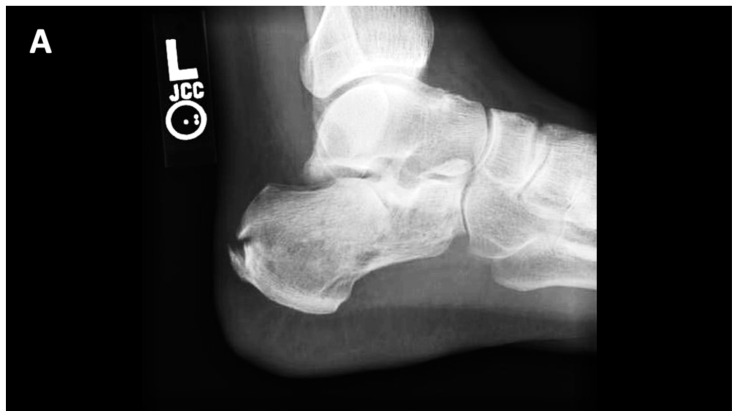
Please see associated 2CLUE2.pptx PowerPoint file

**Figure S1:**
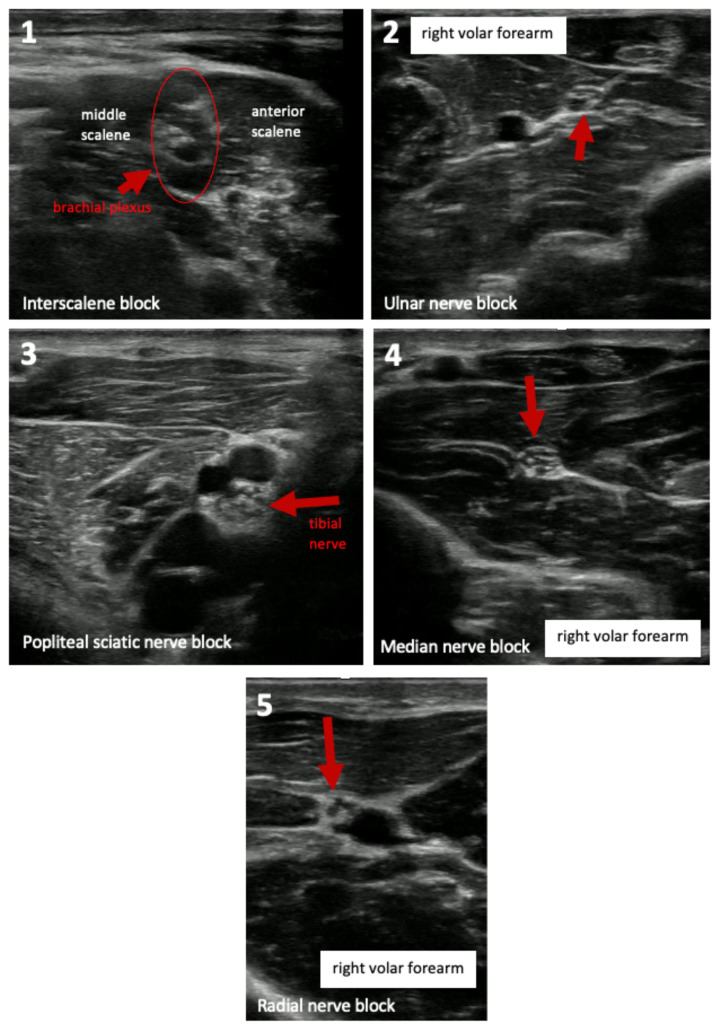
Facilitator’s reference for CLUE 2: Answer key for sonographic anatomy shown. All images are author’s own.

**Figure:**
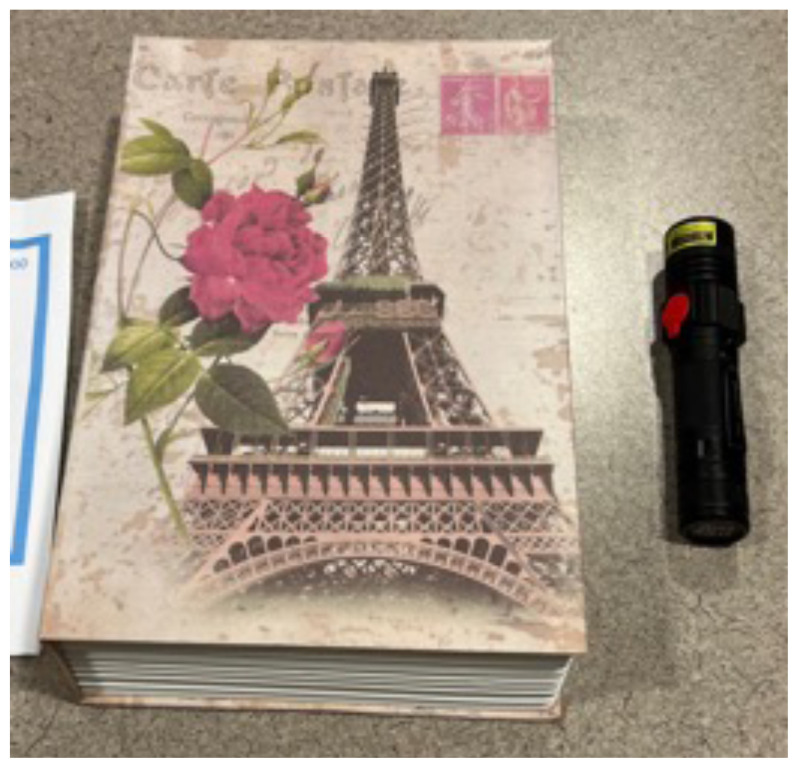
Commercially available lockbox disguised as a book, containing a hand-held UV flashlight. Authors own photograph of example commercially available products.

**Figure 3A, 3B, 3C:**
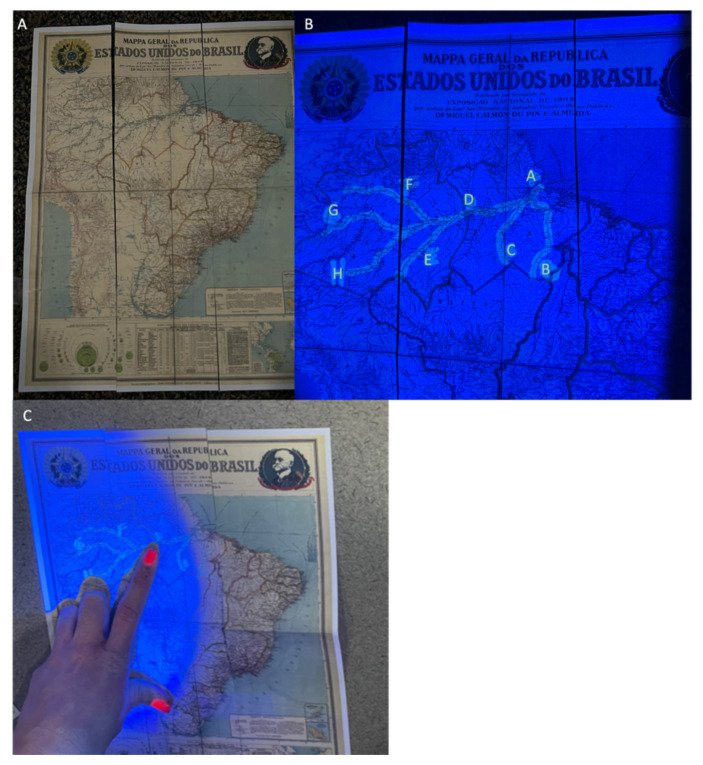
Map cut vertically into three equal strips and marked using UV marker, first tracing the relevant branches of the Amazon River, and then marking the branches A through H to indicate their order from ocean to tributaries (overlayed with text on figure for clarity). Author’s own photographs.

**Figure:**
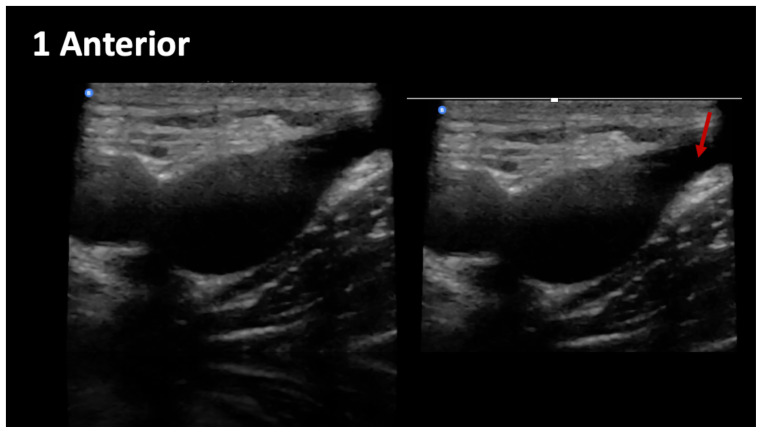
Please see associated CLUE3a.pptx PowerPoint file

**Figure S2:**
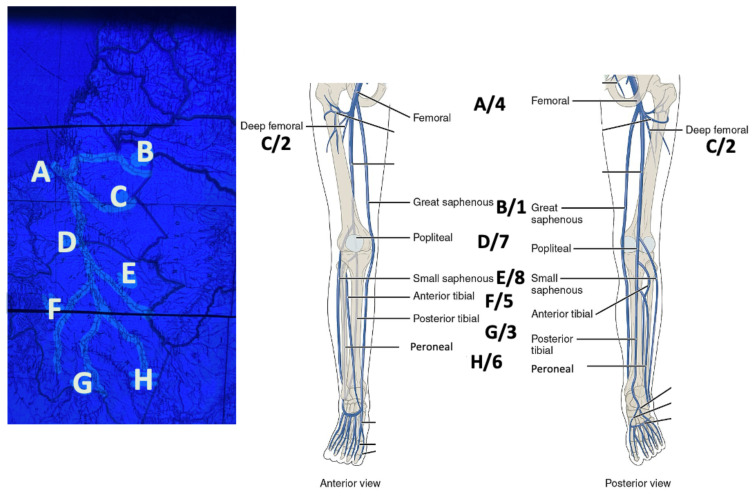
Facilitator’s reference for Clue 3: Matching Brazil map (left pane) to lower extremity venous anatomy (right pane). Letters on map correspond to letters on confluence or vein shown, with number shown corresponding to slide and passcode digit from “ESCAPE” PowerPoint prompt. For example, location “A” on the map is the most proximally encountered structure, which on a standard lower extremity deep vein ultrasound would correspond to the common femoral vein shown on slide 4; therefore, the first digit of the passcode is “4.” Location “B” on the map is the second most proximally encountered structure or confluence, which on a standard lower extremity deep vein ultrasound would correspond to the saphenous vein shown on slide 1; therefore, the second digit of the passcode is “1.”

**Figure:**
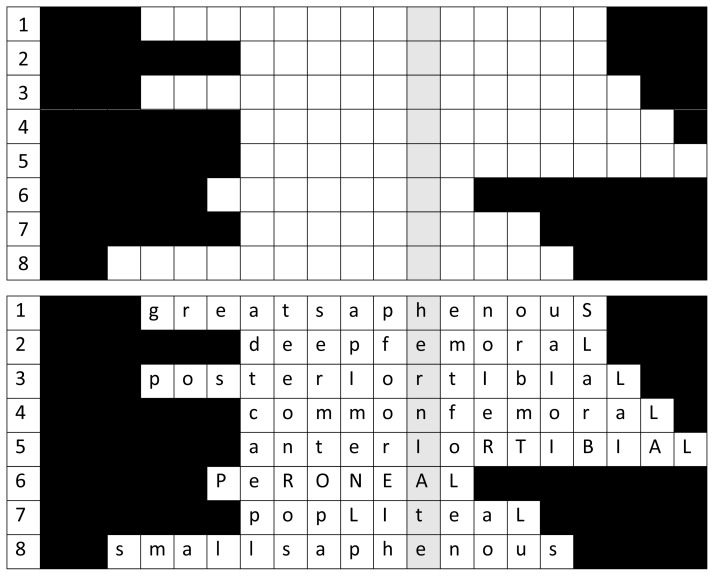
Final puzzle unsolved (above) and solved (below). Vertical gray column indicates the escape password. Please see associated CLUE3bPUZZLED8.dox Word file

## Appendix A : Small Group Application Exercise

### Overview

A successful escape room requires advance planning and preparation. Any room can be used, but a small conference space or unused office is ideal to control the contents of the room (as opposed to an office with personal effects). The game is set before participants enter the room by the session leader. Just before entering the room, participants are oriented to the “theme” of the game, the rules, and their objective. Ideally, participants are permitted to progress through the game with minimal input from the session leader. A set number of hints can be given by the session leader if the team has gotten stuck; additional hints can be given at the discretion of the leader (suggested hints provided in sections below). Some degree of failure (ie, trial and error) is expected, but with hints all teams should be able to progress to the end of the game.

### Preparation

#### Materials List

1. IF using Clue 1 (see Tips for Success)
  - POCUS device with linear transducer
  - Three small plastic cups (must have small enough mouth that linear transducer footprint will NOT fit inside)
  - Clear plastic cup larger than opaque gelatin cup (must have large enough mouth to fit linear transducer inside)
  - Three sealed water bottles or pitcher of cold water
  - Opaque commercial gelatin mix [alternative: clear gelatin, add food colorant]
  - Six coins (2 quarters, 2 nickels, 2 dimes)
2. Computer with PowerPoint and USB port (alternately, load files in advance on computer)
3. Printed color map (from https://www.loc.gov/item/2001620472/)
4. Locking fake book [alternative: envelope] (such as https://www.amazon.com/gp/product/B07D6HFNQZ/ref=ppx_yo_dt_b_asin_title_o04_s00?ie=UTF8&th=1)
5. USB flash drive (unless loading files in advance)
6. UV flashlight (such as https://www.amazon.com/gp/product/B0B97K83J4/ref=ppx_yo_dt_b_asin_title_o03_s00?ie=UTF8&th=1)
7. UV protective goggles or glasses
8. UV marker (such as https://www.amazon.com/gp/product/B01F6IPK00/ref=ppx_yo_dt_b_asin_title_o03_s01?ie=UTF8&psc=1)

#### Starting Items

Participants are introduced to the game (read “Orientation Prompt”) and presented with an empty cup and cups of gelatin (filled with a dime, nickel, and quarter), three sealed bottles of cold water, a flash drive (contents described below) and one piece of a map of the Amazon River Basin (from https://www.loc.gov/item/2001620472/) printed in color. The map should be prepared ahead of time using an ultraviolet (UV) marker as shown below and cut into three segments. The other two pieces of the map can be hidden in the room (for example, taped to the bottom of a chair).

1. **Clue 1 (if using, see tips for successful implementation):** Fill three plastic cups ¼ full with commercially-available gelatin mix (follow package instruction) made opaque with food coloring and allow the gelatin to set. Note: The cup must be small enough that the linear transducer will not fit inside. Once set, place either a quarter, dime, or nickel (one in each container) on the hardened gelatin. Add more gelatin to the cups to fill to ¾ full, covering the coins. Allow the gelatin the set at least 24 hours before use. **Do not freeze gelatin** because this will create air bubbles.
2. **Clue 2:** Load files on flash drive, or load files electronically on computer in the room
3. **Clue 3:** Set the lock box code to “3 – 1 – 2” and place the UV flashlight inside; hide the book somewhere in the room. Print the map in color. Using the UV marker, trace the Amazon River and its specified branches as shown in Figure 3B. Add letters along the river as shown. Cut the map into three pieces and hide two of them in the room. The remaining piece will be given to participants at the start of the game.

#### Orientation Prompt

The facilitator will read the following to the learners: “This escape room will require you to work together as a team to solve three clues to ‘escape.’ The theme of this escape room is ‘in too deep.’ To win the game and ‘escape,’ you must present the correct non-numerical password (up to three tries are allowed). You will have 50 minutes. **Scan the containers to determine the value of the first passcode.**”

### Clue 1: Foreign Body Identification

Note: Clue 1 was tested as described, but is optional and only recommended for advanced learners experienced with escape room mechanics (see tips for successful implementation). Clearly visible on the table or desk are three containers partly filled with commercially available gelatin (with food coloring added to make the gel opaque) which each contain a coin.

Containers should be prepared ahead of time as described. (Note: Make it clear to the team that they are not to destroy or remove the cup contents if this is attempted. For multiple groups back-to-back, author recommends that separate cups should be prepared for each group due to the possibility of melting) The team should be prompted that they should “**scan the containers to determine the value of the first passcode**,” with the monetary value of the contents corresponding to the first two-digit passcode (ie, 40 cents = 40). No ultrasound gel should be provided; instead, the team should deduce they should fill the larger cup with the water to create a water bath for the container (Figure 1B). Only a linear probe with probe cover (to protect the probe) that fits inside the empty cup but not the gelatin cup should be provided, to force the learners to use a water bath. The learners will likely not know how wide each type of coin should be, but they can place the coins given at the start of the game in water and measure them against the coins found in the gelatin to determine their identity. **Table S1** is reserved for the facilitator’s reference and lists the diameters of the coins used.

**Table S1:** Facilitator’s reference for CLUE 1: List of possible foreign bodies (United States coins) and their corresponding diameter. Available from: https://www.usmint.gov/learn/coins-and-medals/circulating-coins/coin-specifications.

Coin Type (value)	Diameter (mm)
Penny (1 cent)	19.05
Nickel (5 cents)	21.21
Dime (10 cents)	17.91
Quarter (25 cents)	24.26

### Clue 2: The Flash Drive

The two-digit passcode (**40**) opens a file on the flash drive (**2CLUE2.pptx**). In the file, there are three X-rays and a series of POCUS video clips. The clips display various nerves and are labeled from 1–5. X-Ray images are labeled A, B, and C and depict a calcaneus fracture, proximal humerus fracture, and 5^th^ finger fracture. Participants should match the correct nerve or plane to the correct location for performing the relevant ultrasound-guided anesthesia technique for the injury shown. For example, X-Ray B shows a proximal humerus fracture, with the corresponding ultrasound image showing the brachial plexus at the level of the scalene muscles. Because this ultrasound image is labeled “1,” this is the second digit of the second unlock code (**312**). Figure S1 provides a reference for the facilitator.

### Clue 3: The Map

The second unlock code (**312**) opens a lockbox disguised as a book hidden in the room (alternatively an envelope held by session leader) which contains a UV flashlight (Figure 2). (Note: It should be stated to participants that protective eyewear should be used upon receiving the UV flashlight.) The map should be prepared ahead of time using UV marker with locations along the Amazon River marked A–H (Figure 3A, Figure 3B); participants will need to find the two hidden parts of the map in the room and put them back together to solve Clue 3.

The flash drive contains an unlocked file (**CLUE3a.pptx**) with a series of POCUS clips of the lower extremity veins. Each clips shows a discrete, identifiable sonoanatomy location in the lower extremity venous system. Participants should match the clip shown to the location on the map based on the river’s branch/confluence points (ie, the most proximal confluence point encountered in a standard lower extremity duplex exam is the common femoral vein and saphenous vein confluence). The order of the clips will provide an 8-number passcode when matched to the letters on the map from distal (ie, outflow into the ocean) to proximal river (**41278536**). Note: Some residents may be unfamiliar with the more distal branches such as anterior tibial vein, posterior tibial vein, peroneal vein, and small saphenous vein – these should be familiar to ultrasound fellows, but additional hints may be needed depending on the mix of learners (ie, if learners have identified all other branches, it would be reasonable to give them the remaining names if they get stuck here). Figure S2 provides a guide to Clue 3 for facilitators.

Left pane adapted from: Companhia Lithographica Hartmann-Reichenbach, Calmon M. *Mappa geral da Republica dos Estados Unidos do Brasil*. In: The Library of Congress, 1908. The materials in The United States and Brazil: Expanding Frontiers, Comparing Cultures are in the public domain or have no known copyright restrictions and are free to use and reuse. https://www.loc.gov/item/2001620472/

Right pane adapted from: Lower Limb Veins Anterior Posterior. In: Illustration from Anatomy & Physiology, Connexions, 2013. Creative Commons Attribution 3.0. http://cnx.org/content/col11496/1.6/

### Clue 3b: The Crossword

To meet the objective of being able to name each vein based on the displayed sonoanatomy, this third and final numeric passcode (**41278536**) will unlock a third file (**CLUE3bPUZZLED8.docx)** on the flash drive that contains a single diagram as described below (Figure 4). When listed by name (ie, “great saphenous”) in order from 1–8, the relevant letters are underlined, and this reveals the password to escape (**HERNIATE)**.

### Sample Hints List (will depend on where learners get stuck)

1. The number in the file name is the number of digits in the password to open the file (i.e., CLUE3bPUZZLED8 has an 8-digit password).
2. The LOST2 password comes from the gelatin cups and should be solved first.
3. There are three pieces to the map. (If learners did not find the book or map pieces, hints can be given to find them.
