# Supplementary material for: In Too Deep: A Point-of-Care Ultrasound (POCUS) Escape Room
Source: J Educ Teach Emerg Med. 2025 Oct 31;10(4):SG50–66. doi: 10.21980/J8.52100 (PMC12594470; doi:10.21980/J8.52100)
Supplement: Supplementary file 1 [file 10-4-SG50-CLUE2.pptx]

## Slide 1
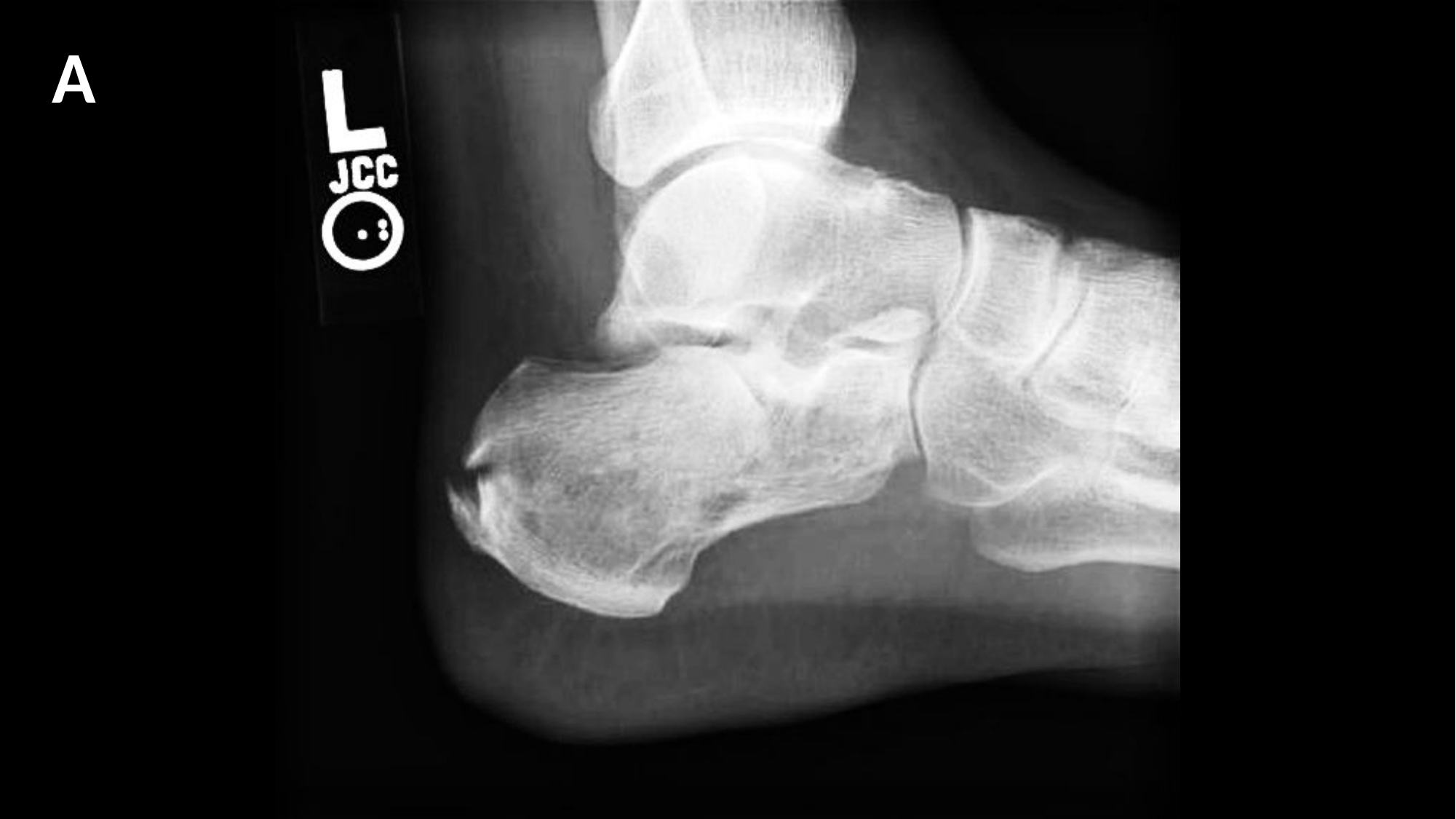

A

## Slide 2
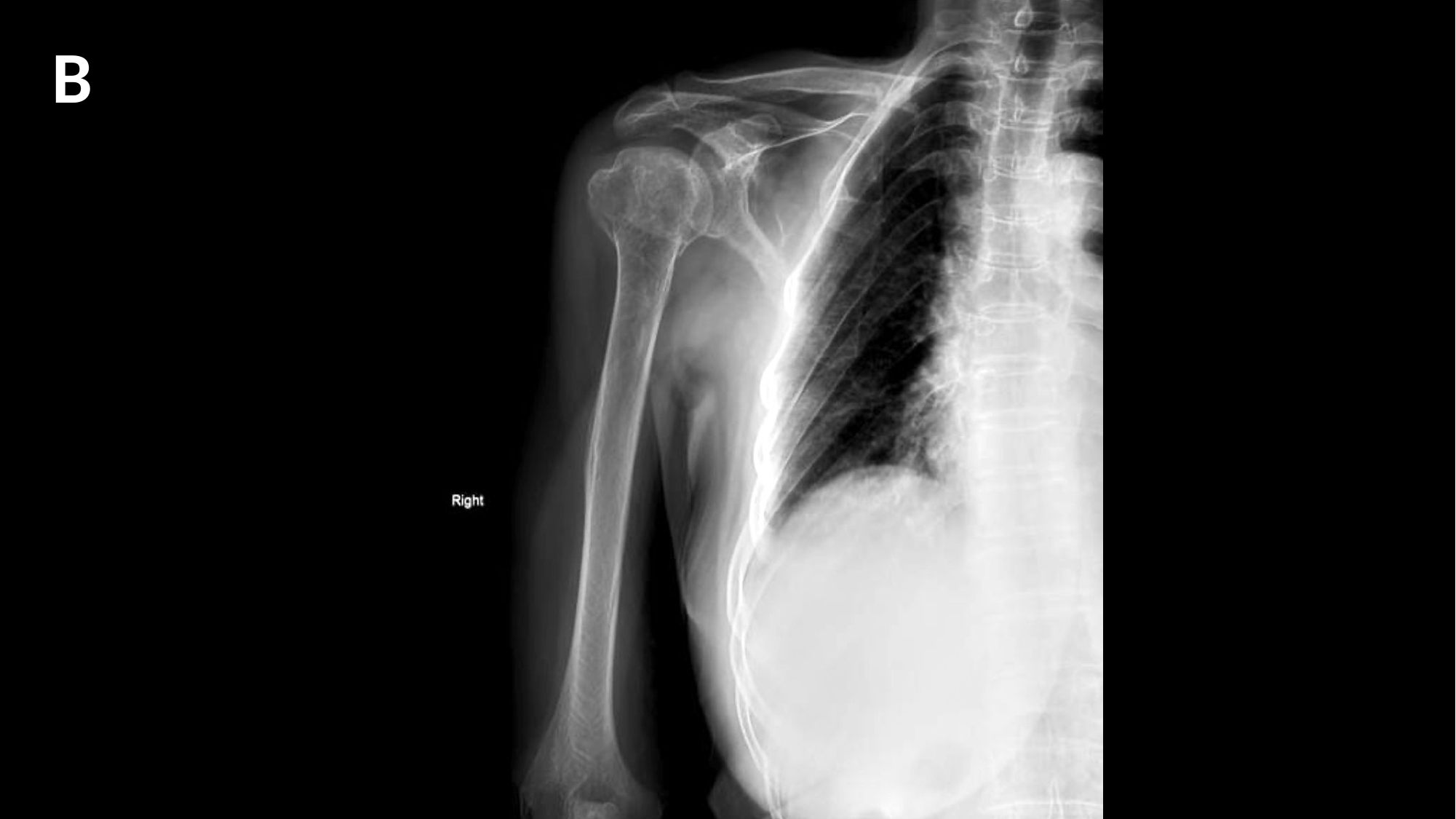

B

## Slide 3
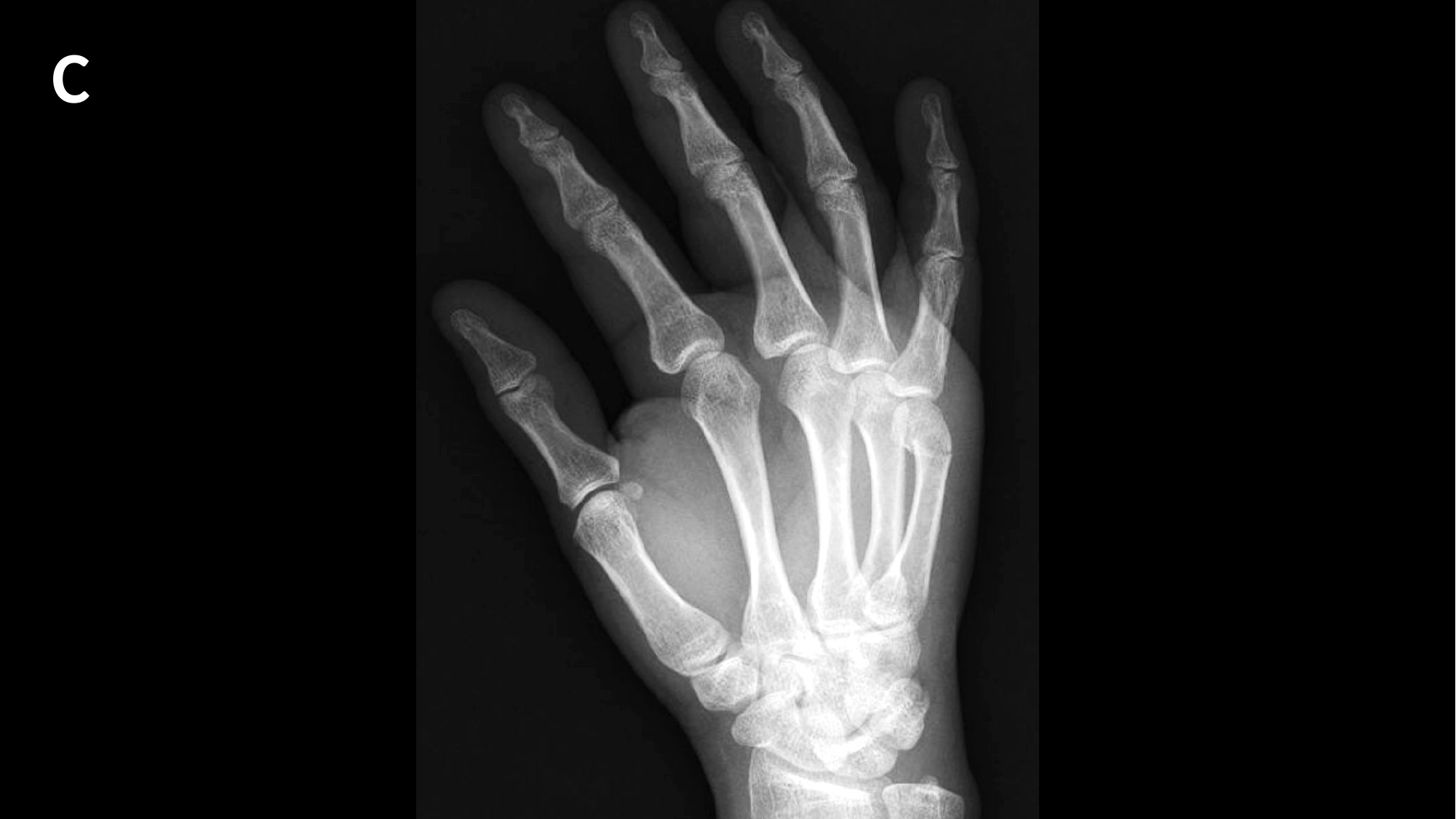

C

## Slide 4
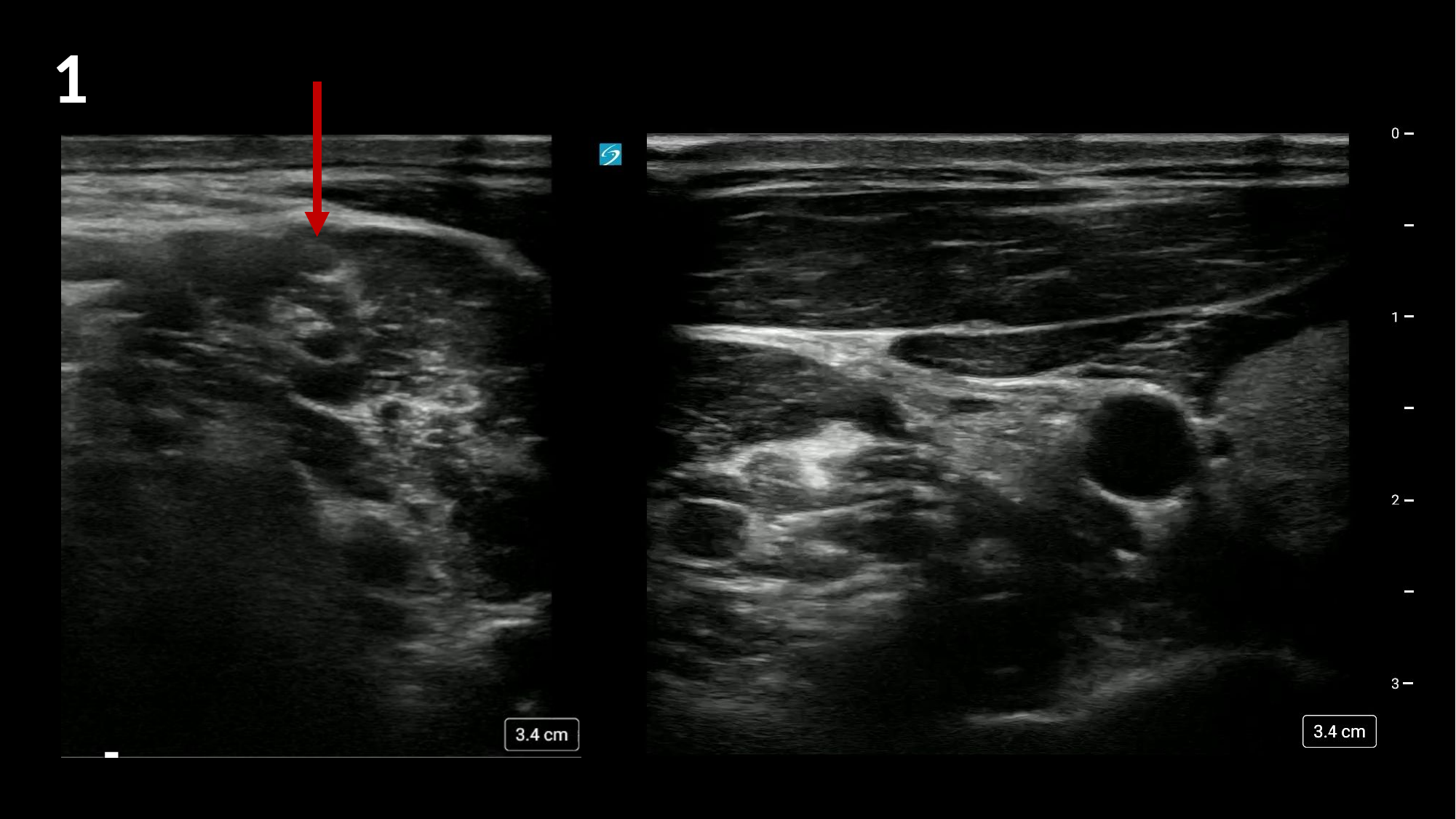

1

## Slide 5
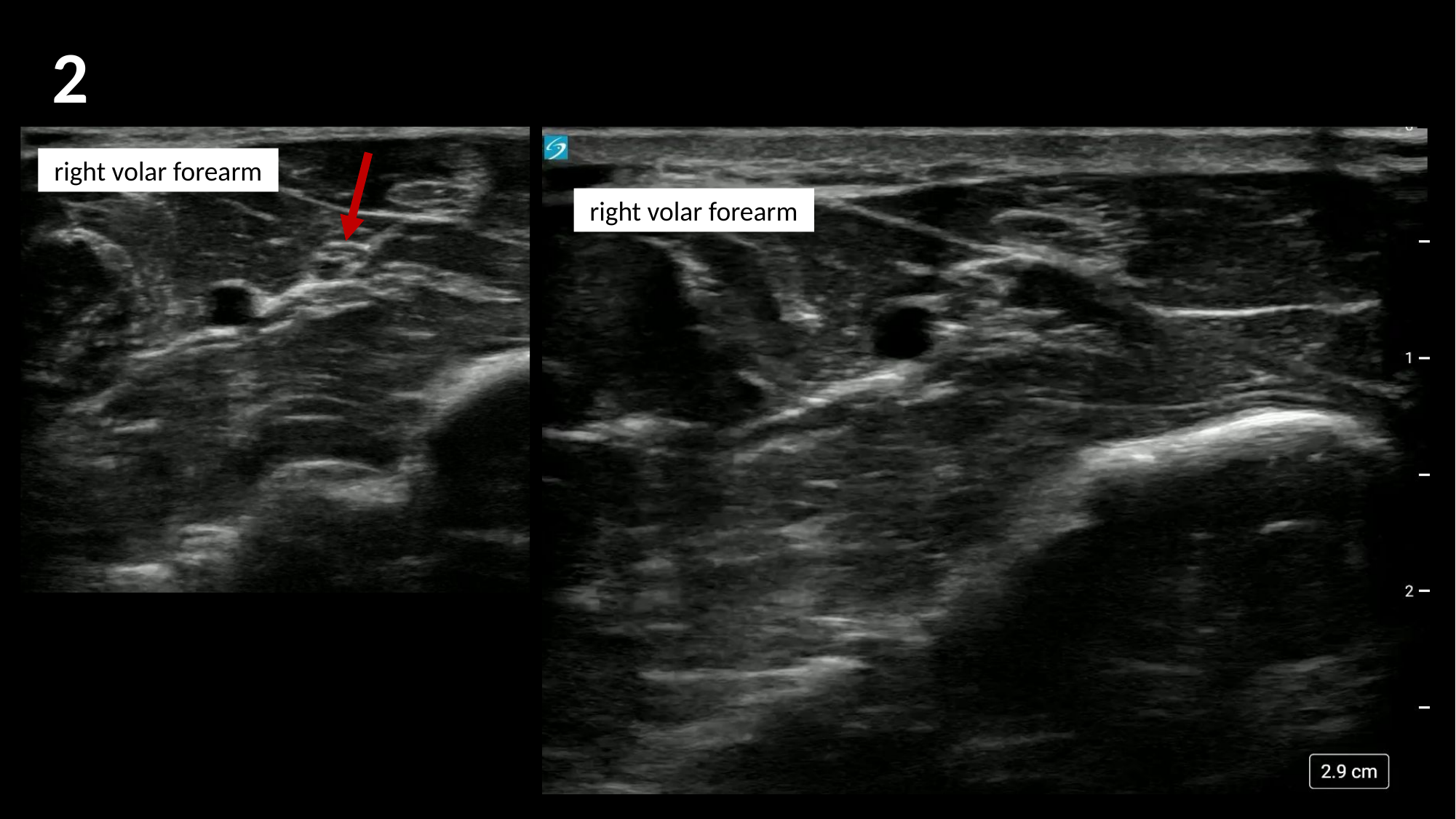

2
right volar forearm
right volar forearm

## Slide 6
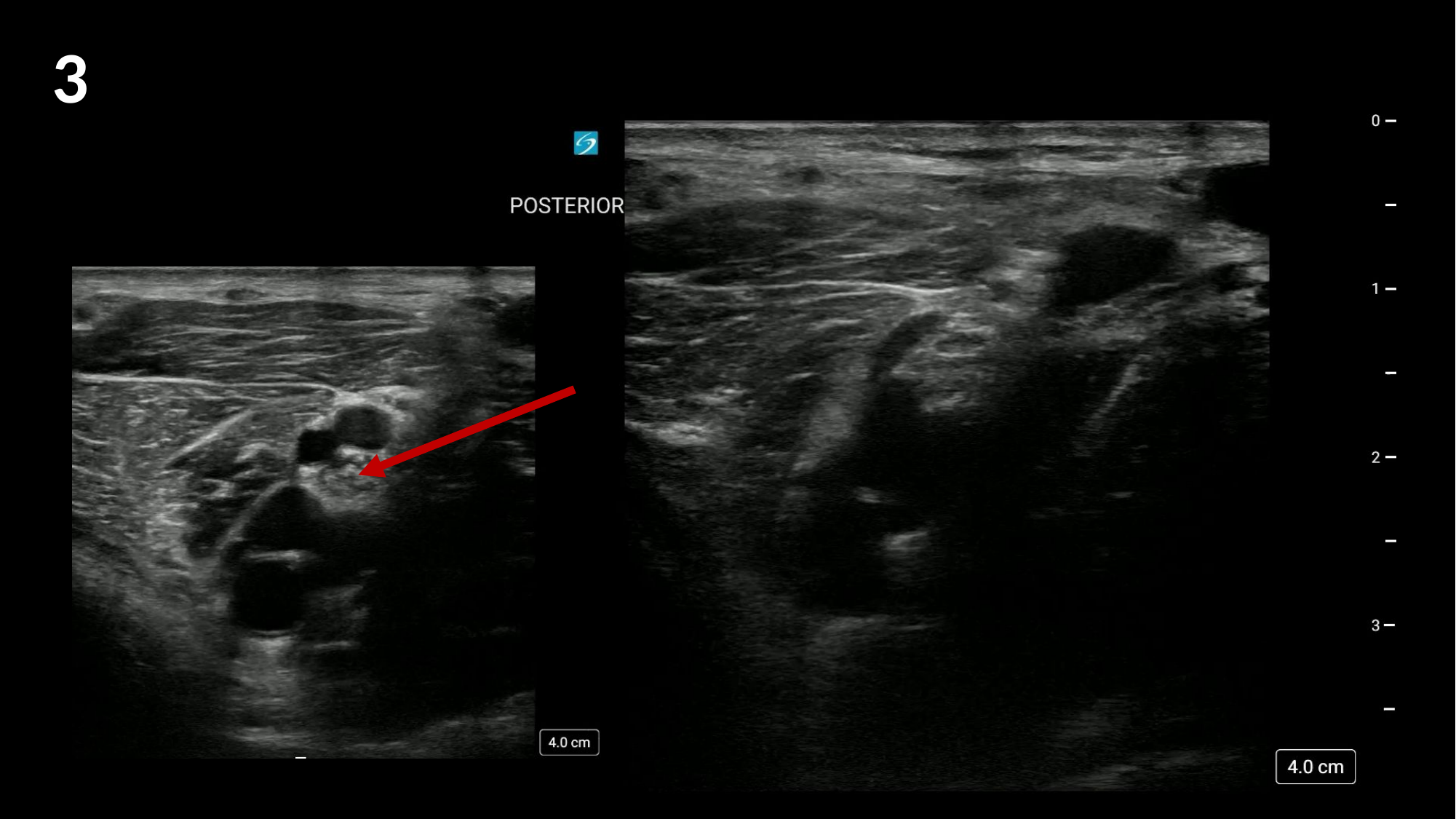

3

## Slide 7
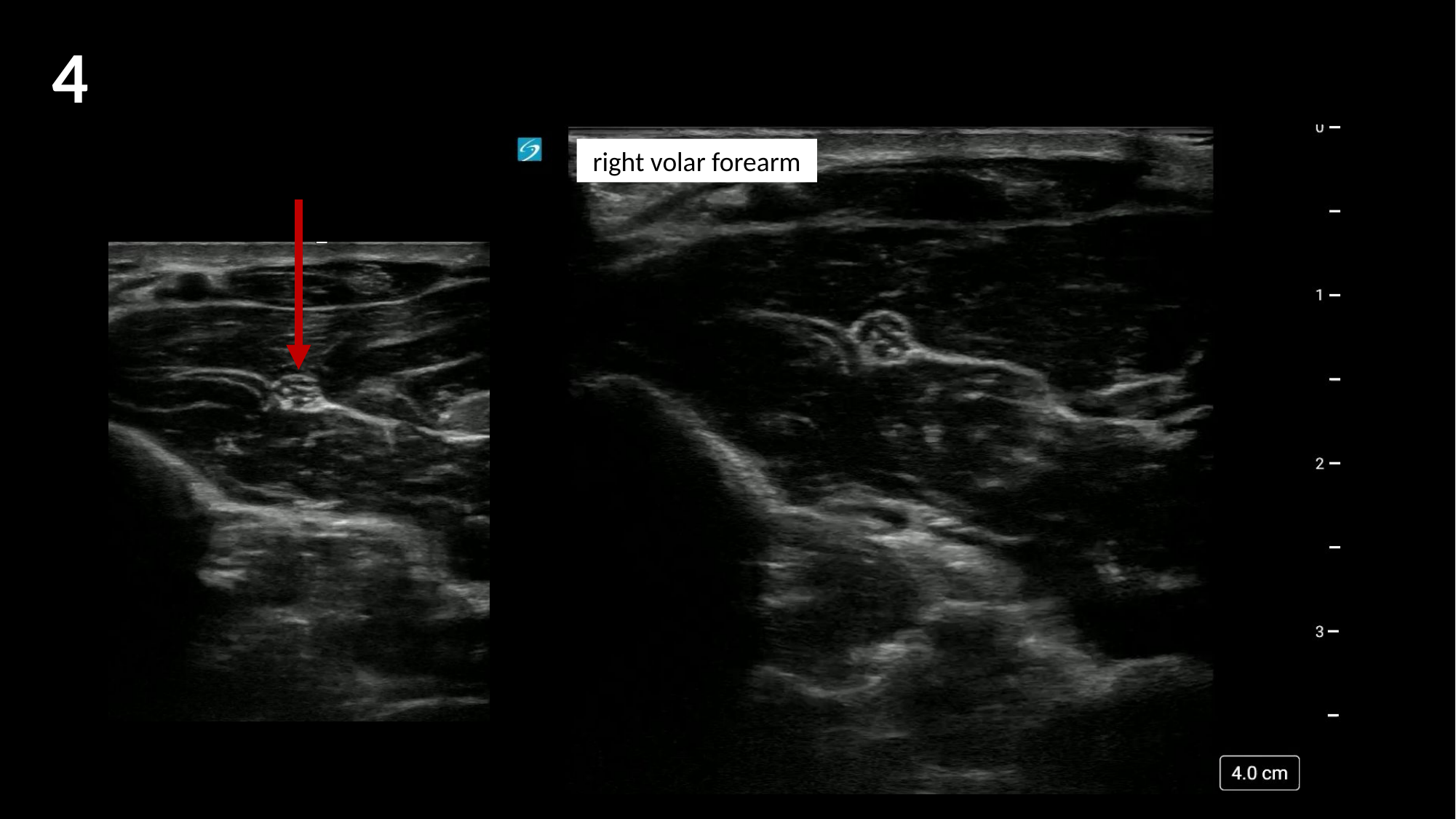

4
right volar forearm

## Slide 8
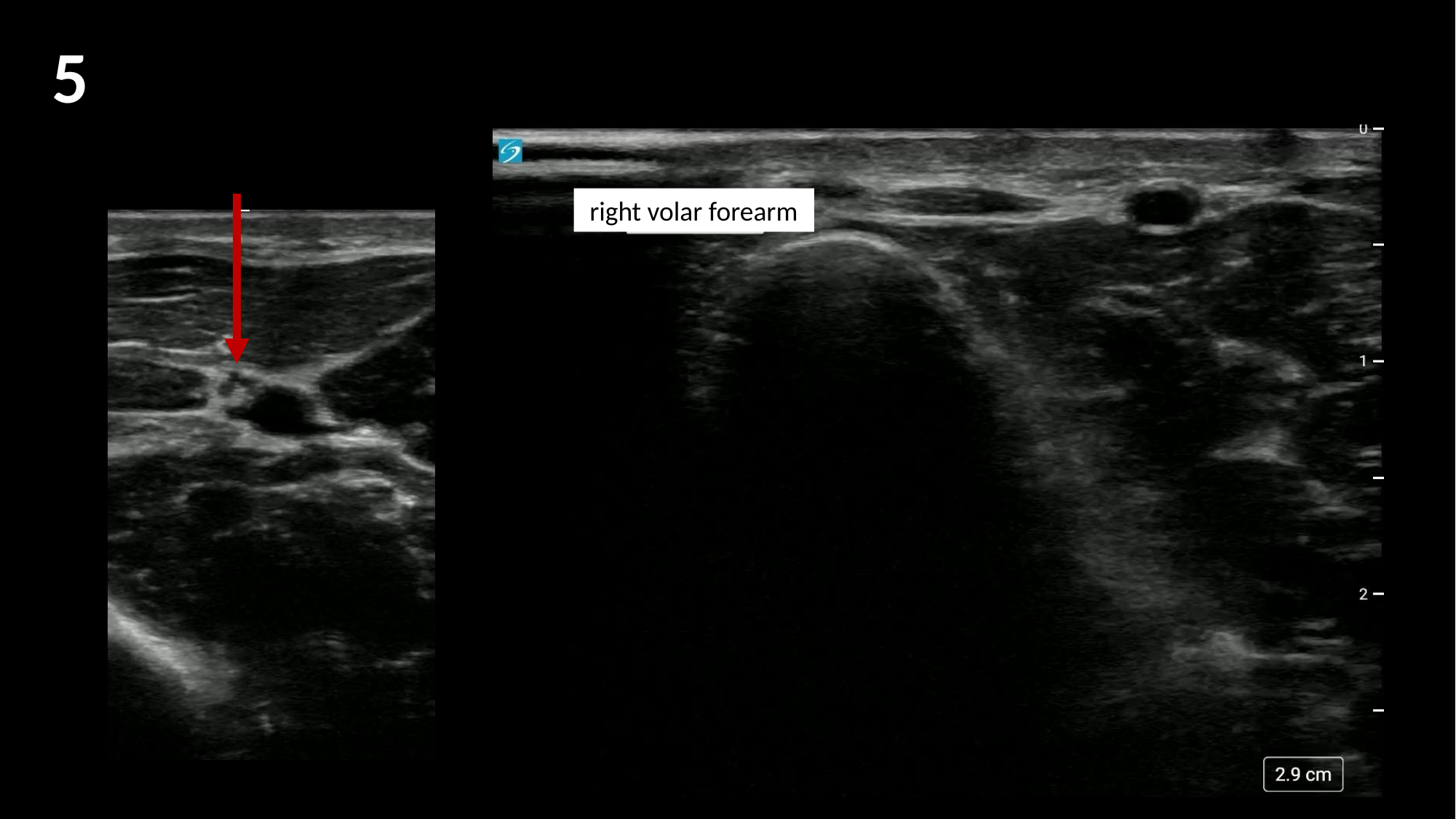

5
right volar forearm
