# Supplementary material for: In Too Deep: A Point-of-Care Ultrasound (POCUS) Escape Room
Source: J Educ Teach Emerg Med. 2025 Oct 31;10(4):SG50–66. doi: 10.21980/J8.52100 (PMC12594470; doi:10.21980/J8.52100)
Supplement: Supplementary file 2 [file 10-4-SG50-CLUE3a.pptx]

## Slide 1
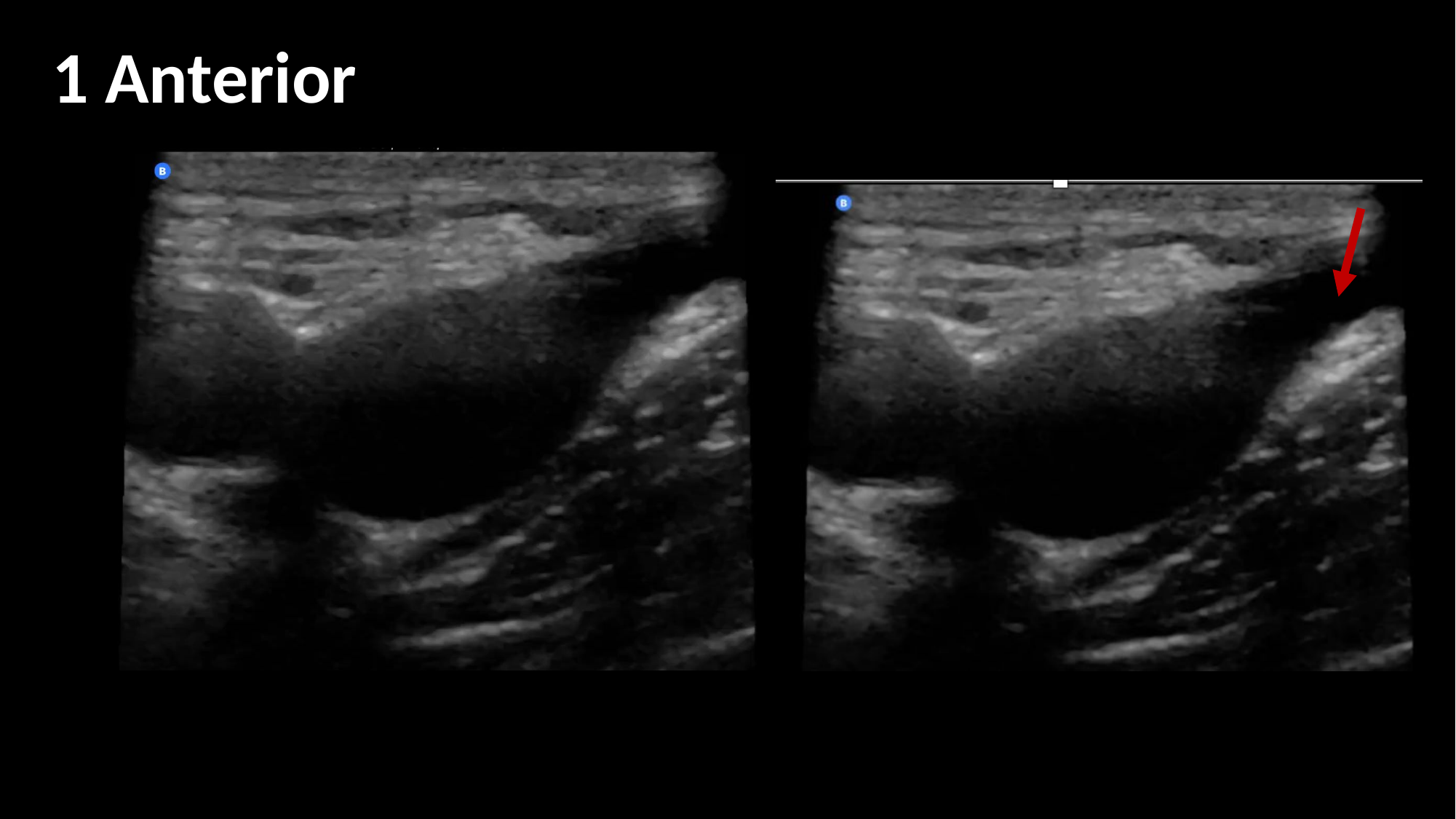

1 Anterior

## Slide 2
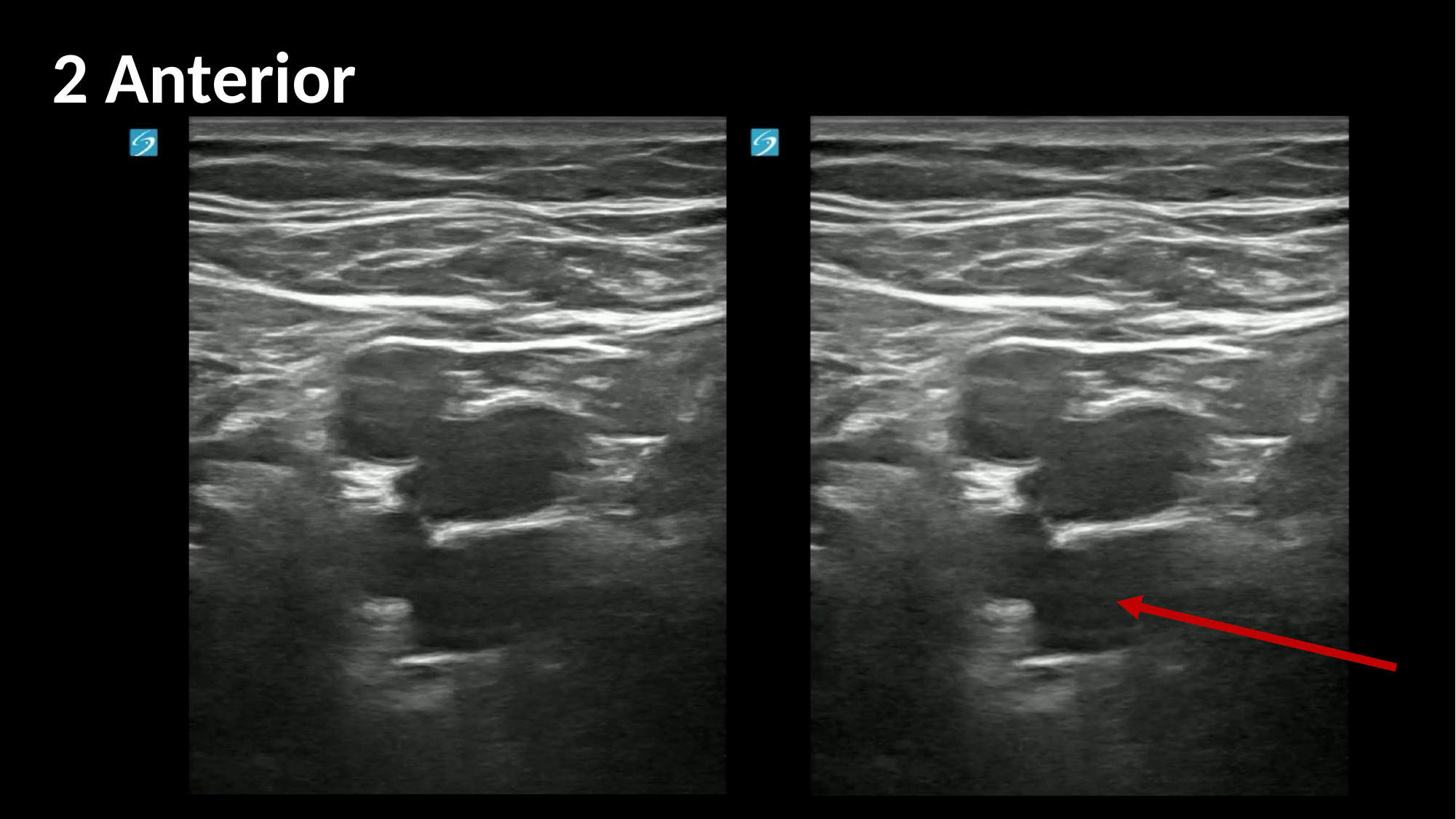

2 Anterior

## Slide 3
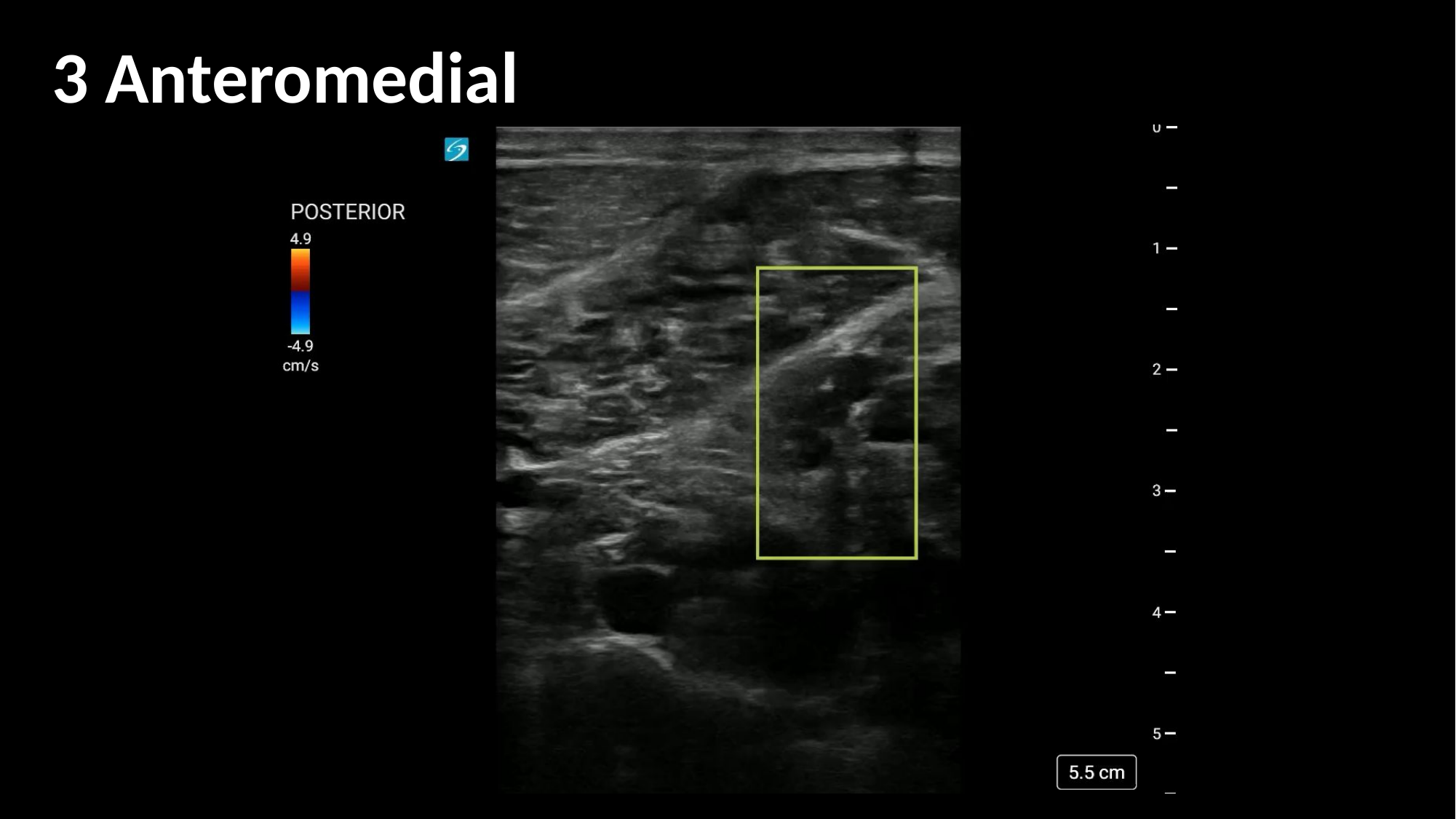

3 Anteromedial

## Slide 4
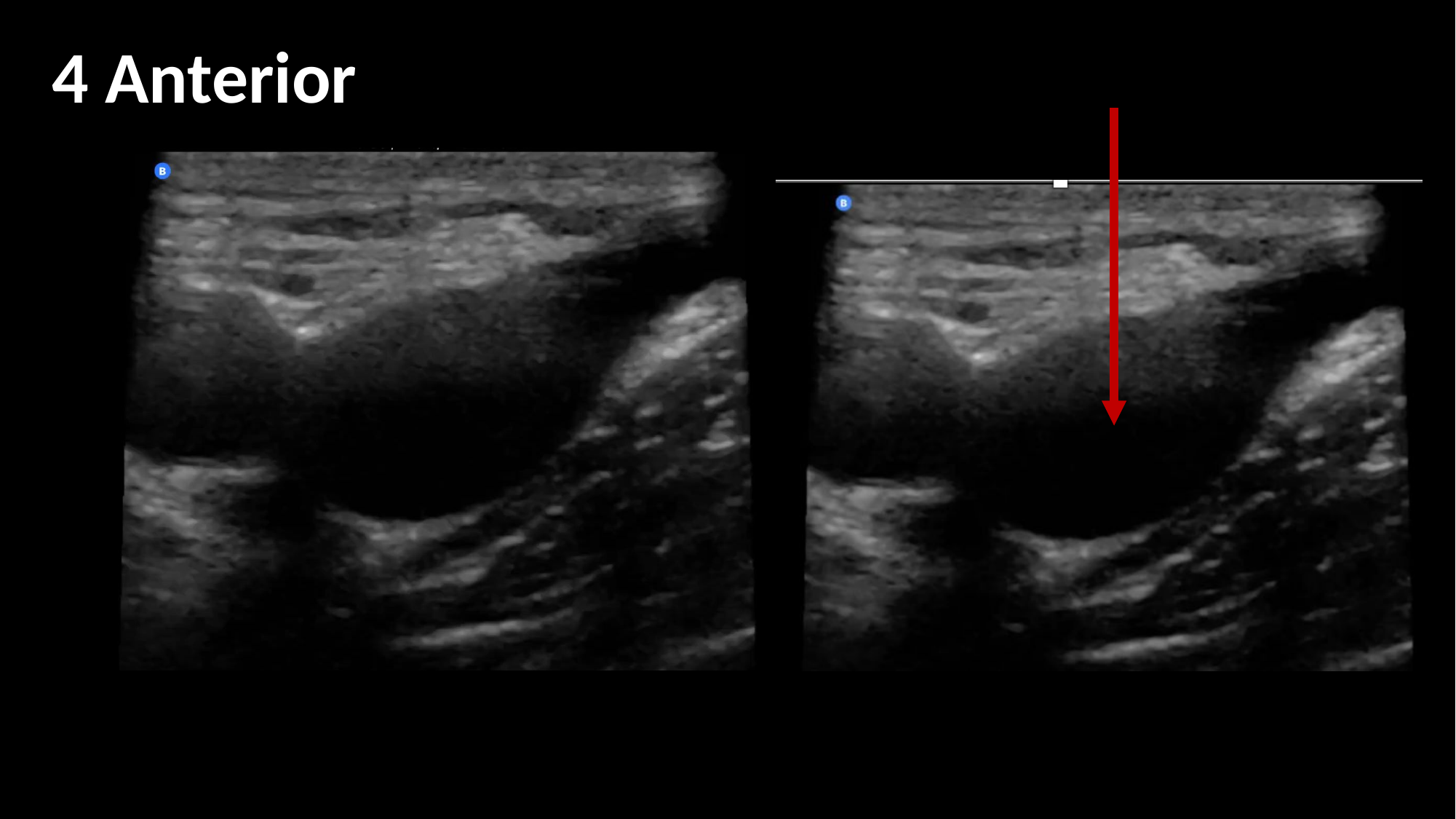

4 Anterior

## Slide 5
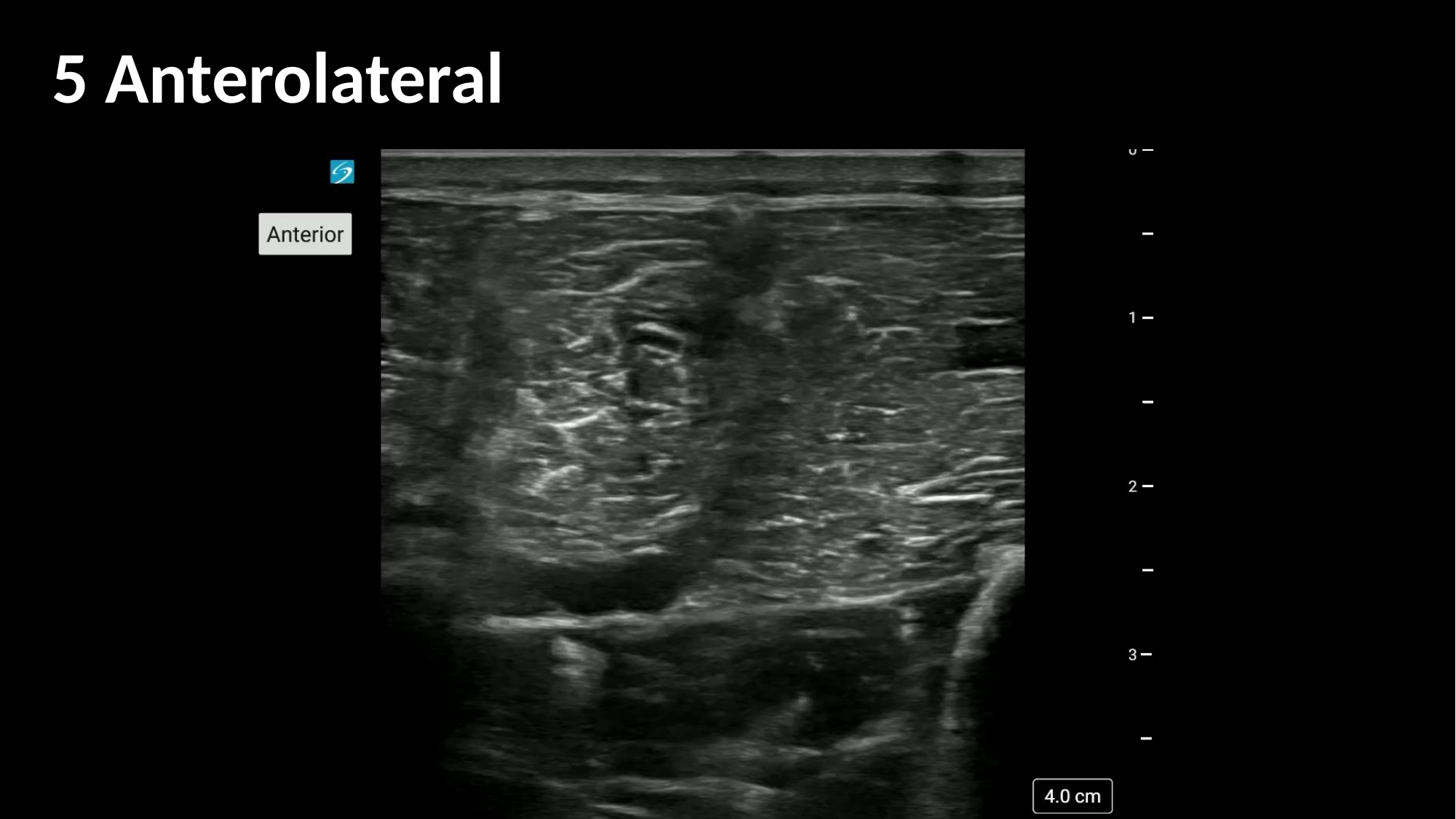

5 Anterolateral

## Slide 6
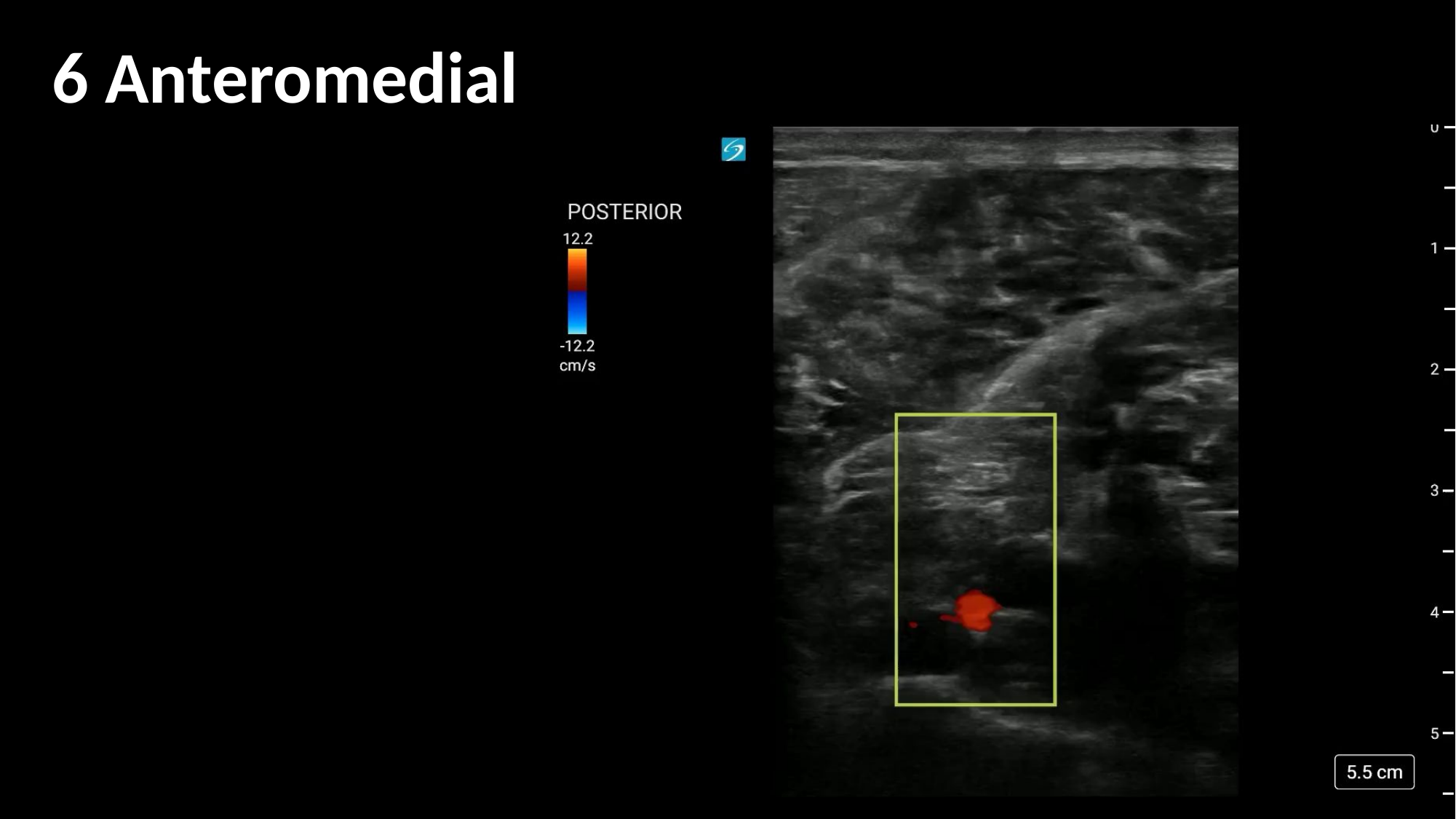

6 Anteromedial

## Slide 7
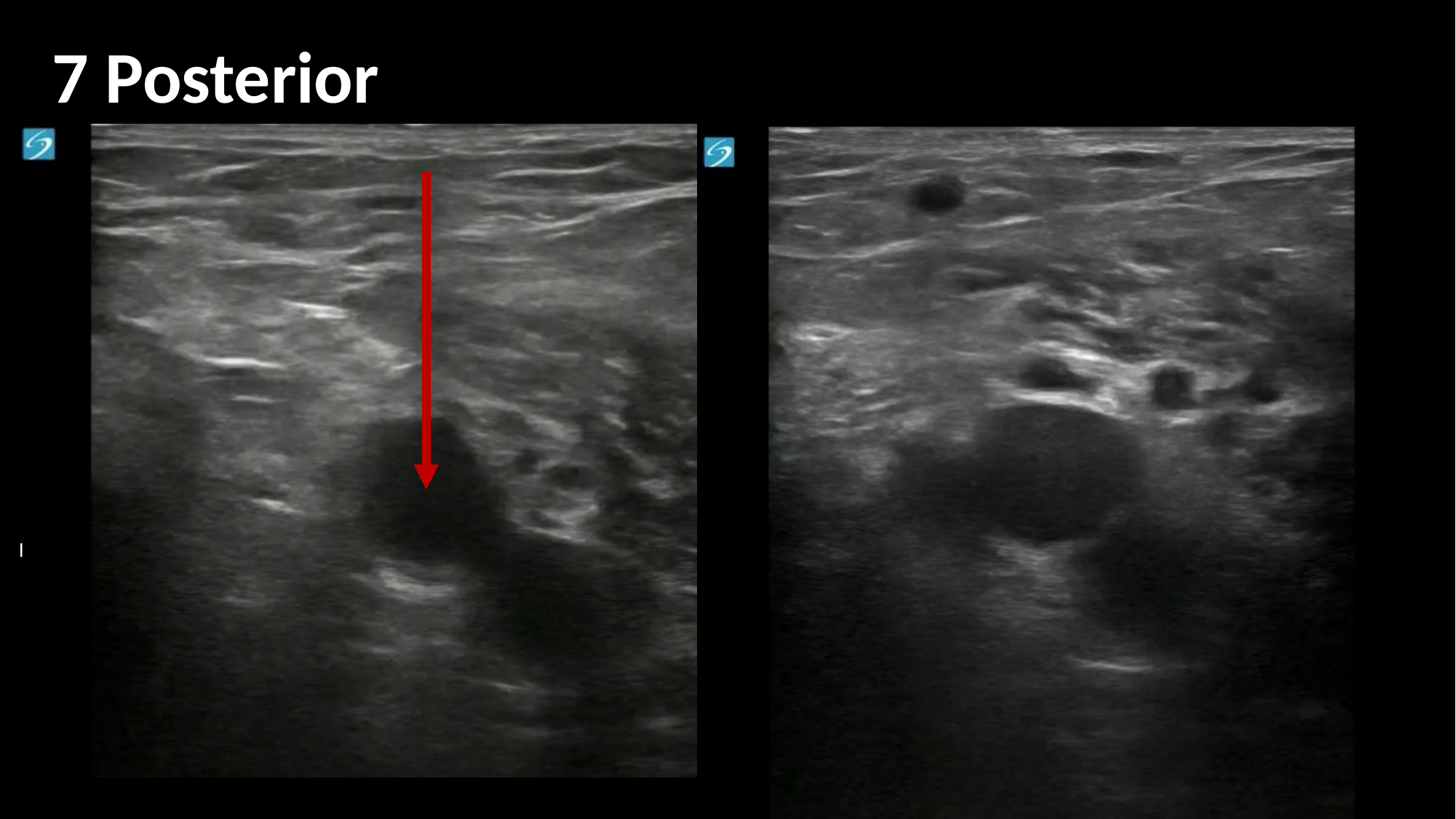

7 Posterior

## Slide 8
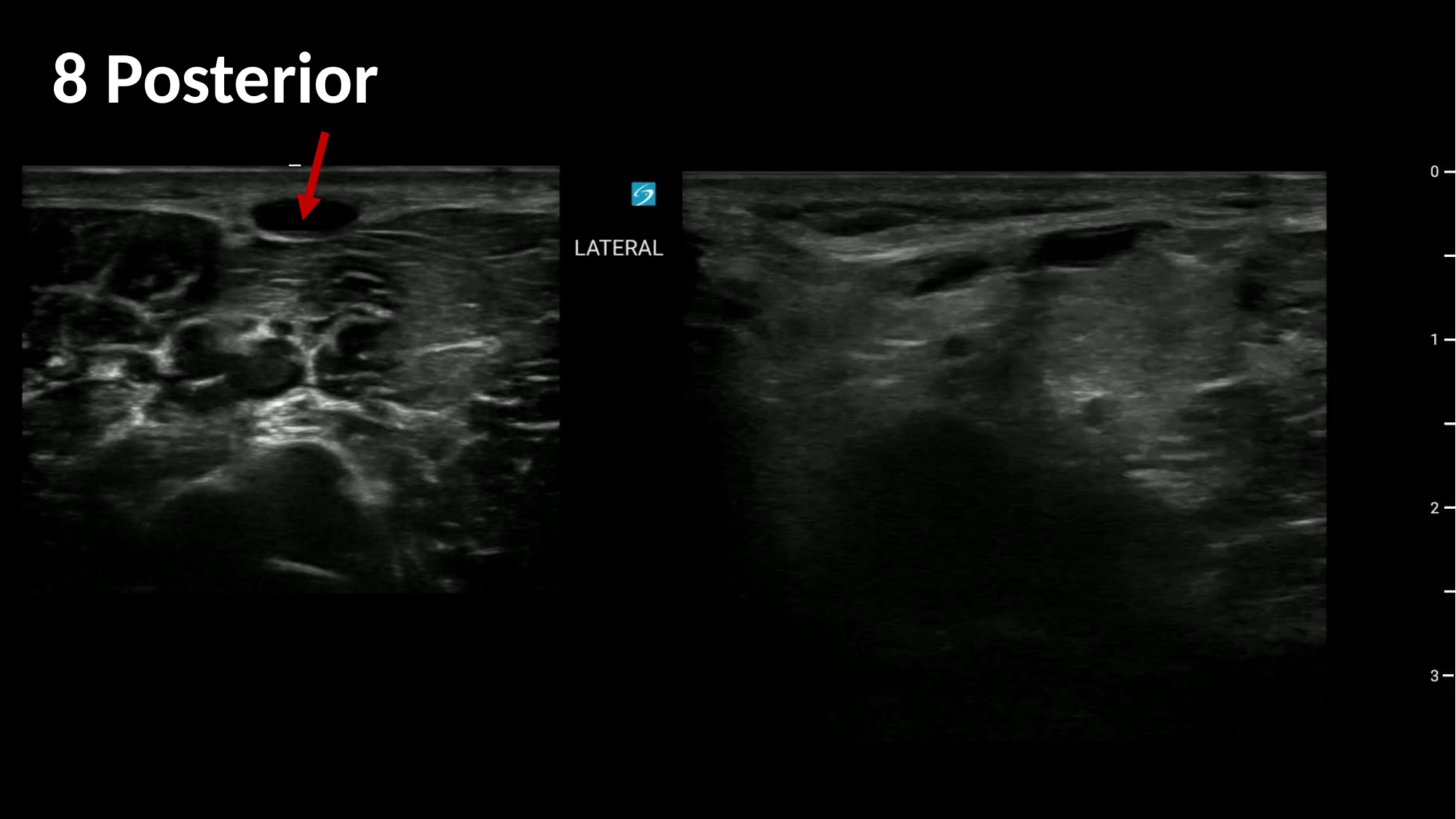

8 Posterior
