# Supplementary material for: In Too Deep: A Point-of-Care Ultrasound (POCUS) Escape Room
Source: J Educ Teach Emerg Med. 2025 Oct 31;10(4):SG50–66. doi: 10.21980/J8.52100 (PMC12594470; doi:10.21980/J8.52100)
Supplement: Supplementary file 4 [file 10-4-SG50-Supp1.docx]

All images are author’s own.
